# Informal Safety Communication of Construction Workers: Conceptualization and Scale Development and Validation

**DOI:** 10.3389/fpsyg.2022.825975

**Published:** 2022-03-17

**Authors:** Weiyi Cong, Hong Xue, Huakang Liang, Yikun Su, Shoujian Zhang

**Affiliations:** ^1^School of Civil Engineering, Harbin Institute of Technology, Harbin, China; ^2^School of Management, Shandong University, Jinan, China; ^3^School of Economics and Management, Beijing Jiaotong University, Beijing, China; ^4^School of Civil Engineering, Northeast Forestry University, Harbin, China

**Keywords:** informal safety communication, scale development, intrinsic motivation, factor analysis, Chinese

## Abstract

Existing studies have highlighted the importance of informal safety communication among workers at construction sites. However, there is still a lack of empirically tested theoretical models with valid and reliable scales for describing and measuring construction workers’ informal safety communication (CWISC). Accordingly, this study aimed to fill this need by developing an instrument to assess the communication performance of construction workers. Four stages of scale development were described: construct formation, item generation, factor extraction through the exploratory factor analysis (EFA) (*n* = 219), and scale assessment through the confirmatory factor analysis (CFA) (*n* = 156). Using questionnaire data drawn from construction workers in China, the CWISC was verified to be a three-dimensional construct including citizenship safety communication (CSC), self-needed safety communication (SSC), and participatory safety communication (PSC). The corresponding CWISC scale with 12 items was shown to have acceptable internal consistency reliability, as well as content, convergent, and discriminant validity. The CWISC scale could serve as an instrument to assess and identify the weaknesses in informal safety communication performance of construction workers. In turn, this information could help supervisors implement appropriate management practices to those workers to enhance workplace informal safety communication. Related studies taking a multidimensional CWISC into account were expected to be carried out.

## Introduction

Despite constant efforts for safety management over the past few decades, construction safety has not been improved as much as compared to other industries, it still witnesses a high rate of accidents (Zaira and Hadikusumo, 2017; Liang and Zhang, 2019). From the perspective of accident investigation, workers’ unsafe behaviors were identified to be the primary and immediate cause of accidents considering that nearly 80% of on-site accidents were caused by unsafe human behaviors (Choi et al., 2017; Li et al., 2017; Zhang et al., 2019). Continued efforts to deepen understanding and decrease unsafe behaviors are still urgently needed.

Safety communication has been regarded as potential intervention for construction workers’ unsafe behaviors (Cigularov et al., 2010). It is not just a process of exchanging safety information and knowledge at the workplace (Liao et al., 2014), but involves effects on workers’ behaviors and their perceptions of safety (Shuen and Wahab, 2016). Safety communication was discussed in many studies for its significant potency in high-risk work environments (Zohar and Luria, 2003; Chan et al., 2016; Albert and Hallowell, 2017). From the perspective of information flow, safety communication can be carried out in three directions, upward, parallel, and downward (Yates and Orlikowski, 1992). Considering the role played by it in these situations, when detecting potential hazards and learning specific safety rules to adhere (Parker et al., 2001; Shuen and Wahab, 2016; Pandit et al., 2020), improving safety communication was suggested to be an essential measure in achieving fewer safety incidents on job sites (Vecchio-Sadus, 2007; Kines et al., 2010; Liao et al., 2014). Workers constitute the largest group on construction sites, and homogeneity of their identities reasonably increases the tendency of safety communication within crews (Turner et al., 2010; Allison and Kaminsky, 2017). Parallel safety communication, an action between subjects with the same position, was more likely to promote safety behaviors compared with leader–worker interactions (Zhou et al., 2008; Lingard et al., 2019). Given that the importance of leader–worker safety communication had been supported and highlighted in existing studies (Huang et al., 2018; Su et al., 2019), parallel safety communication between workers needs more attention.

The communication structure of a successful organization should include both formal and informal communication. Construction workers’ informal safety communication (CWISC) was defined as safety-related information sharing through channels outside pre-established structures of an organization (Alsamadani et al., 2013). It is a personal activity held at work place with no arranged agenda, and takes the typical form as an impromptu safety conversation based on the current exposures that may be urgent and alarming (Tang et al., 2015). Specific dialog situations can be “A worker passes by another worker and reminds about a past hazardous event to avoid its reoccurrence,” and “A worker asks another worker for advice on safety operation.”

In contrast to formal safety communication preplanned at a fixed time as pre-construction safety trainings or toolbox talks (Rajendran et al., 2009; Hallowell, 2012), CWISC provides a more flexible channel, which is not limited by time and place, this preponderance contributes to convey safety information in a timely manner (Wu et al., 2017). When workers are caught in information ambiguity triggered by perceived risks in the process of operation, the action of seeking safety information from coworkers leads them to respond safely. The importance of CWISC has solicited attention from scholars in safety research areas, a few attempts were made to explore its characteristics and impacts on safety performance. According to a comparative research on the social network conducted by Alsamadani et al. (2013), the crew in which workers had numerous informal safety communication links presented a lower injury rate, and the closely linked crew showed an increased capacity to manage potential errors before they lead to an incident (Alsamadani et al., 2013). Further, using a modified questionnaire developed by Alsamadani et al. (2013), Allison and Kaminsky (2017) reported that workers in mixed-gender crews relied more heavily on informal communication for their safety information (Allison and Kaminsky, 2017).

Although the importance of CWISC in construction safety has been recognized, its core characteristics remained unclear, a lack of a well-designed (i.e., reliable and valid) measurement for assessing multidimensional CWISC was observed. In most existing studies, the existence of different channels (formal and informal) had been recognized, but they did not make a distinction when measuring it. For instance, Pandit et al. (2020) introduced both formal and informal safety communication among construction workers, but then all of these descriptions were reclassified into “safety communication” to be measured (Pandit et al., 2020). Limited studies focusing on measuring CWISC employed the method of social network analysis (SNA), which was argued to be inaccurate and error-prone (Fischbach et al., 2009), remained a vacancy in the measurement scale. In addition, practices of different types of CWISC were available in recent studies. Allison and Kaminsky (2017) described CWISC as “calling friends in another crew on how to handle a work problem” (Allison and Kaminsky, 2017), while Kaskutas et al. (2016) gave an example of it as “teaching dialog between experienced workers and other workers” (Kaskutas et al., 2016). The purpose for initiating the aforementioned safety communication was obviously different; nevertheless, the CWISC was generally treated as an unexpanded variable, and the categories and corresponding motivations of it remained unclear.

Being one of the exploratory studies on this issue, this article aimed to give a particular focus on identifying specific dimensions of CWISC and develop a well-designed instrument to describe and assess it. The research drew upon the insights of the safety communication literature, and engaged in a four-stage process to systematically explore and examine the measurement scale.

### The Present Research

The goal of this research is to construct and validate an efficient measuring tool of CWISC capturing different dimensions. To reliably and accurately assess a theoretical construct, a systematic and rigorous process of development and validation should be followed (Farooq, 2016; Kim et al., 2021). A deductive–inductive approach with mixed methods (qualitative study and quantitative study) was applied to study the issue, by which both summative derived categories and those correlations randomly arisen from data may find their way into this study (Itzkovich, 2021). The approach was appropriate as there was little theory to guide notions about specific forms of CWISC (Yang et al., 2018).

**FIGURE 1:**
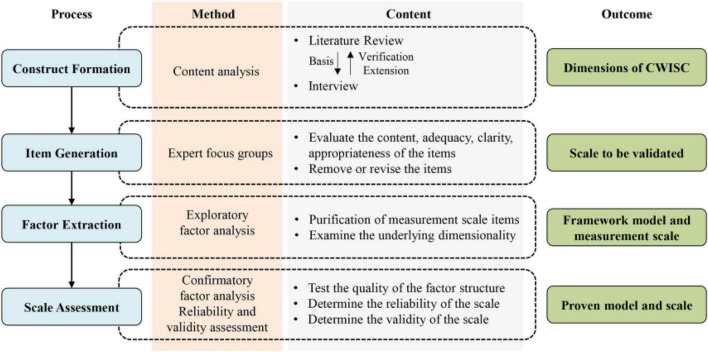
Development process of the construction workers’ informal safety communication (CWISC) scale.

For the development of a framework and the corresponding scale of CWISC, four activities were conducted with the help of voluntary construction workers in China; namely, construct formation, item generation and content validity assessment, exploratory factor analysis (EFA), and confirmatory factor analysis (CFA). The overall flow with relevant methods and specific contents embedded in the research was illustrated in Figure 1.

## Study 1

### Methods

#### Literature Review

Literature review, one of the qualitative research methods, was first applied to broaden insights into CWISC. Through a holistic summary, potential compositions of the construct can be used as a starting point to clarify the focus of the next stage. In this process, practices of safety communication and descriptions of related theories were collected from an open resource, then a series of pre-established categories were proposed (Hinkin, 1998; Yang et al., 2018).

Based on a systematic analysis of the potential terms, a three-level keyword structure, adaptively modified from Liang’s search strategy (Liang and Shi, 2021), was adopted to cover diverse and large-scale search terms for comprehensively and reliably obtaining CWISC-related articles (as shown in Table 1). The context keywords defined the search context, which was limited to the construction industry; the topical keywords further narrowed the search scope; and the subject keywords limited the search subject to construction workers.

**TABLE 1:** The used three-level keyword structure.

Search levels	Retrieval strategies
Context keywords	TS = (“construction industr*” or “construction work*” or “construction compan*” or “construction organization*” or “construction project*” or “building project*” or “construction site*” or “construction management” or “construction activit*” or “construction task*”)
Topical keywords	TS = (“safe* communication*” or “safe* information*” or “communication* safe*” or “safe* voice*” or “safe* exchange*” or “safe* interact*” or “safe* discussion*” or “risk* communication*”)
Subject keywords	TS = (employee* or worker* or coworker* or labo* or carpenter* or apprentice*)

“*” Denotes the fuzzy search strategy that is used to capture the variation in terms. “TS” represents the topic research strategy where an article is included when required terms are identified in any positions of the title, abstract, and keywords.

The Thomson Reuters’s Web of Science (WoS) core collection database was selected because it is a source of standardized and high-quality academic publications. By restricting the literature type to article, a total of 78 candidate articles were obtained. To further eliminate the literature outside the target topic, abstracts were screened seriatim according to the criterion of minimizing information missing. After a careful screening process, a total of 37 CWISC-related articles were identified for the following literature review. The literature excluded involved the following themes: the development and application of emerging safety-related technology (BIM, virtual reality, wearable devices), safety communication in other industries, and safety communication between other participants (leaders and workers, management departments).

#### Semi-Structured Interviews

##### Participants

Recorded documents, participative observation, and interviews are all typical methods for obtaining data in qualitative research. However, workers’ safety communications on construction sites are normally not recorded by the organization, and remote participative observation has limited access to the specific content of communication. Given this, semi-structured interviews were selected to excavate exact communication performances.

A number of semi-structured interviews to safety managers from several construction projects and workers within their jurisdiction were carried out in person, then a match between theoretical literature and practical conditions was checked to further bridge the gap. In total, 26 workers participated in the interview voluntarily from March 2021 to April 2021. To reduce self-reporting and recalling biases of workers, three safety managers and five supervisors, each with more than 5 years of safety management experience, were also invited to interview. Face-to-face and online interviews were selectively applied according to a comprehensive consideration of geographical accessibility and participants’ wishes.

##### Procedure

**FIGURE 2:**
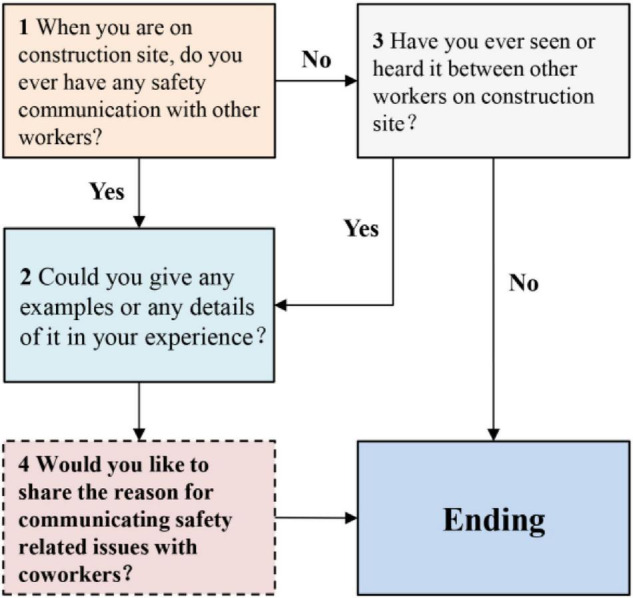
The designed interview process.

Before interviews, CWISC was introduced to participants to give them a clear understanding about the concept, the action intensified the compatibility and validation of answers. Abide by the semi-structural interview syllabus, questions were executed in accordance with the following designed process (Figure 2). In addition to questions about workers’ performances during safety communication, the reasons for carrying out or engaging in these activities were further explored. Open-ended questions were given to workers who have participated in safety communication. The significance of CWISC was affirmed by workers as the interactive process helped them understand safety rules better and behave in a safe manner.

For the interviews to safety managers and supervisors, they were asked for comments on workers’ performances: “Are there private safety communication between workers on construction sites? Could you tell us any details of it?”

##### Analysis

Transcription and analysis of interview recordings were performed by three researchers who mastered the content analysis method. After eliminating ambiguous and irrelevant responses, the following steps were adopted to obtain final classifications. First, keywords were extracted and marked from descriptive texts of safe communication performances and motivations. Then, these keywords were categorized into different themes and further matched with the initial types derived from the literature, this step continued until no matches emerged. Finally, the characteristics of the remaining unclassified themes were summarized and discussed to define new types.

### Results

#### Preliminary Classification From a Literature Review

In terms of research paradigms and method applications, prior studies associated with workers’ safety communication can broadly be split into two categories. The first embranchment took the communication social network as the primary research object, and comparative analyses were carried out to identify correlations between social network characteristics with specific safety performance [i.e., hazard recognition skill (Pandit et al., 2020) and safety climate (Lingard et al., 2019)]. The second embranchment presented empirical studies into safety climate and safety citizenship behaviors (SCB) within workgroups, aiming to reveal a causal mechanism with other organizational and individual indicators (Meng and Chan, 2020; Zhang et al., 2020). Within these studies, workers’ safety communication behaviors were involved in subdimensions of the first-order construct. Surprisingly, empirical studies closely related to CWISC were relatively scarce.

Focusing on the collation of descriptions and measurements, valuable clues had emerged during the extraction process. Firstly, in terms of social network analyses, participants were commonly invited to fill in the names of workers with whom they exchange safety information, together with choosing options on different frequencies and modes, in which informal safety discussion and formal safety exchange (training, toolbox talking, and written communication) were, respectively, installed (Alsamadani et al., 2013; Kaskutas et al., 2016). Secondly, in the domain of safety climate, safety communication was considered to be one of the explanatory indicators of safety climate. Specific communication behaviors were adopted to directly measure the unidimensional safety climate or assigned to a subscale, sample items were “Coworkers remind each other to take precautions” (Zhang et al., 2020) and “Safety issues are openly discussed between my supervisor and my workgroup” (Beus et al., 2019). Further, similar items were applied to measure safety voice behavior, which was widely acknowledged as a subdimension of SCB. Safety voice was defined as an active behavior making constructive suggestions related to safety concerns (Yang et al., 2018). Typical descriptions were “I tell my colleague who is doing something unsafe to stop” and “I discuss new ways to improve safety with my colleagues” (Tucker et al., 2008).

Obviously, the aforementioned safety communication under different interests was endowed with diverse interpretations. Due to the lack of a clear CWISC concept, the understanding of CWISC remained scattered and its subdimensions have not been fully expanded. Facing this challenge, two categories were aggregated grounded in analyses on the intention of communication. “Interpersonal helping,” borrowing from Yang’s definition, was used to describe the communication with helping intention (Yang et al., 2018), while “Safety discussion,” drawing on the mentioned keyword, referred to the communication with an intention of sharing information. Typical statements were sorted as shown in Table 2.

**TABLE 2:** Preliminary classification of construction workers’ informal safety communication (CWISC) from the literature.

CWISC classification	Described scenarios	References
Interpersonal helping	Instructional sessions with an experienced worker that may have a safety focus.	Kaskutas et al., 2016
	Calling friends on how to handle a work problem.	Allison and Kaminsky, 2017
	In this workgroup, coworkers remind each other to take precautions or work safely.	Gao et al., 2017; Zhang et al., 2020
	When my colleagues are in a dangerous situation, I will remind and help them in time.	Meng and Chan, 2020
	My co-workers are quick to point out unsafe conditions.	Beus et al., 2019
Safety discussion	Work-related discussions with co-workers.	Alsamadani et al., 2013; Ho, 2013
	Safety issues are openly discussed between my workgroup.	Beus et al., 2019
	‘I’ve got this issue, do you guys have this issue?’ ‘Yeah, that’s what I’ve had happen to me too, we resolve it by…’	Lingard et al., 2019
	I always discuss with my colleagues about improving safety and reducing the potential risks for the current works.	Tucker et al., 2008; Meng and Chan, 2020
	When you have safety related issue at work, you will discuss it with your colleague and request for assistance.	Chen et al., 2021

#### Construct Formation

Given the greater amount of colloquial expressions, much time was spent on transcribing and summarizing the main ideas of workers. After removing ambiguous descriptions, such as communication actions with indeterminate time and place, the results of interviews came in the form of a 28,000-word interview record and 71 CWISC events. Keywords were summarized and marked to identify the initiator of the communication, the sender, and receiver of the information, the time, and purpose of the activity and the feeling of the communication, this process was conducted by taking the sentence as the unit of analysis. Considering that safety communication activities generally include basic elements, such as communicator, communication time, and safety information, a single keyword cannot be used as the basis for the identification and classification of safety communication activities, but a combination of keywords within a statement. Then, based on the keywords, 9 themes were generated and categorized into subdimensions of CWISC, the cumulative results of coding for CWISC were shown in Table 3, the results were verified, the proposed two types of CWISC were enriched, and further a third type was developed.

**TABLE 3:** The results of coding for CWISC.

Affiliation category	Themes	Concurrent keywords	Frequency	Samples of original statements from interviews
SSC	N1: Sending a self-protection signal	Information sender; active participation; self-safety; working time	6	N4 I am a scaffolder, once I was on the high scaffolding in the work, my coworker was going to carry out the work of downward adjustment of scaffolding, I hastened to signal my workers to stop and wait for me go down from the scaffolding.
	N2: Consulting to coworkers	Information receiver; active participation; self-safety; working time	9	N7 My parents and child depend on me, so I usually pay much attention on safety issues, I always consult the coworker or supervisor promptly when there is not clear on my work. N18 Once the supervisor asked me to take over the work of a worker, for safety purposes, I consulted with the worker in detail about the situation that had occurred in his previous operations.
	
CSC	N3: Informing coworkers of safety rules	Information sender; active participation; coworker’s safety; explanation; working time	4	N03 When a new worker came to the crew, I took him to familiarize with the site and explained to him the safety rules to follow.
	N4: Reminding coworkers to notice safety	Information sender; active participation; coworker’s safety; remind; working time	7	N15 Once when we were working, there was a safety hazard, I rushed to tell other workers to pay attention to.
				N25 A worker told me that he found his coworker had little safety awareness, he often reminded him at the construction site.
	N5: Being asked about safety advice	Information sender; passive participation; being asked; working time	10	N29 A worker found that the electricity line was slightly aging, he asked me if it could continue to use, he thought there would be no problem generally, I told him to immediately report to the supervisor and replace it.
	N6: Being reminded of safety matters by coworkers	Information receiver; passive participation; being concerned; working time	6	N19 While I was doing my work, a coworker told me to watch out for the high-tension line.
	
PSC	N7: Listening to others working experiences	Information receiver; discussion; rest time	5	N20 A worker told us a dangerous situation occurred previously which he had seen, he was scared after that, he reminded us to always be safe in our work.
	N8: Discussing with coworkers	Information exchanger; discussion; rest time	16	N12 Once a few of us workers discussed the meanings of a warning sign on site, the supervisor explained to us finally. N30 On several occasions, I saw workers chatting on site together, I learned through inquiries that they were sharing their experiences in safety operation.
	N9: Sharing during discussion	Information sender; discussion; rest time;	8	N6 I am willing to share my experiences when discussing safety issues with my coworkers.

Because there was a lack of sufficient understanding on CWISC, the original name of aforementioned dimensions was drawn on keywords of existing studies. With the in-depth investigation of workers’ performances and corresponding motivations, a rational perspective was expected to be followed so that they can be normatively and synchronously renamed. Intrinsic motivation, reported as a critical predictor of behaviors, was employed as a principle of naming. Finally, safety communication represented by “safety discussion” was categorized as participatory safety communication (PSC); citizenship safety communication (CSC) was used to summarize communication behaviors for the purpose of “interpersonal helping”; self-needed safety communication (SSC), a newly developed category, included communication behaviors of seeking self-protection.

##### Citizenship Safety Communication

In this study, the concept of CSC was first introduced for those altruism-based communication phenomena on construction sites. Similar to the definition of SCB, CSC refers to extra-role safety information exchange through informal channels. It focuses on improving coworkers’ safety performances beneficial to coworkers and organization. Four detailed themes were gathered from interviews, they were “Informing coworkers of safety rules,” “Reminding coworkers to notice safety,” “Being asked about safety advice,” and “Being reminded of safety matters by coworkers.” Further, CSC accounted for 27 out of total 71 events, “Being asked about safety advice” was the most mentioned scope. Of the 27 reported CSC events, 11 were related to the active helping behaviors of communication initiators. In addition, workers reported some communication activities that were not originated by their own. In total, 10 workers claimed that they had been asked by fellow workers to confirm if the operation was safe as well as to explain the safety signs to coworkers.

Two characteristics of CSC were revealed through interviews. The first characteristic stresses its intrinsic altruistic motives of individuals, worrying about the potential injury of coworkers acting as a promoter for originating or participating in CSC. The second characteristic emphasizes that it is a discretionary positive act outside workers’ responsibilities. Supervisors had made it clear that such helping behavior was encouraged but not prescribed, it would not be rewarded by the organization.

##### Self-Needed Safety Communication

The second type was SSC: help-seeking information exchange sought to ensure sponsor’s own safety. During interviews, some workers pointed out the straightforward reason for taking SSC as they needed to keep themselves safe on worksites, which appeared to be an essential characteristic of SSC. Two themes under SSC were motivated by the worker’s safety need; they were “Sending a self-protection signal” and “Consulting to coworkers.” SSC accounted for the least among the three communication behaviors, with only 15 events reported. Of the 15 reported SSC events, 9 were related to “Consulting to coworkers,” others were “Sending a self-protection signal.” These two themes represented different safety information flows. Behaviors of “Sending a self-protection signal” were accompanied by the sending of safety messages, whereas actions of “Consulting to coworkers” were expected to receive safety information.

Compared to the extra-role altruistic features of CSC, SSC is an intra-duty action that workers should perform to achieve their own safety. This important point of view emerged from the explanations of supervisors. They claimed that the organization had provided workers with the necessary safety training and protective equipment; in return, workers were given the responsibilities and obligations to operate in a safe manner. They should take positive actions to achieve safety goals, not only for the organization, but also for themselves.

##### Participatory Safety Communication

The third type, PSC, describes safety communication for the purpose of sharing and exchanging safety-related information. Specific themes were “Listening to others working experiences,” “Discussing with coworkers,” and “Sharing during discussion.” There were totally 29 events associated with PSC, which was the most common form of CWISC.

Some distinguishing features of PSC were generated corresponding to the other two types of CWISC. PSC is first labeled by its analysis-oriented function compared to solution-oriented function of CSC and SSC. The focus of such an action is to share and exchange information with coworkers, it is about their own understanding of safety without an mixed intention for improving their own and their coworker’s safety immediately. As workers described, they mostly carried out PSC during the rest time. The second characteristic stresses the dynamicity of the interactive process, in which the worker can act as both the sender and receiver of safety information.

##### Summative Comparison of Three Subdimensions

These three types of CWISC provided access to transmission and sharing of safety information between workers on construction sites. As communication is a process that requires the involvement of more than two individuals, it can be defined as different types according to motivations of participants. The following scenario is a common communication process, when worker A asks worker B for advice on how to operate in a safe manner, and worker B gives a detailed explanation. Defined from the motivation of A, he initiates SSC, whereas in terms of B, he participated in the CSC passively. A pertinent summarized comparison of the three subdimensions was presented in Table 4 for legible understanding.

**TABLE 4:** A summary of the comparison of three subdimensions of CWISC.

	CSC	SSC	PSC
Motivation	Altruism-actuated	Safety needs	Sharing
Objective	Solve the problem	Solve the problem	Analyze the problem
Responsibilities attributes	Extra-role	Intra-duty	Extra-role
Occurrence time	Under construction	Under construction	Rest interval
Content	Operation-based	Operation-based	Extensive and random

## Study 2

### Methods

#### Procedure

This study followed Evans’s procedure of developing measurement items (Evans et al., 2007), the process of identifying scale items began by reviewing items used in previous measures of SCB, safety climate, and safety voice. Items that were applicable to the themes established earlier (N1–N9) were selected. Some items were included without a change, others were slightly modified to adapt to the construction background. Then additional items were written from interviews to fill in the themes that have not been populated. Items were designed to be general to apply across different trades. An example of a “general” item was “I can freely ask questions to my workmates if I have safety problems.” An example of a “specific” item was “I can freely ask questions to my workmates when I do not know the right place to start taking down scaffolding.” Further referring to the study of Zhang et al. (2020), items of communication perception were used in their study to measure safety communication between the supervisor and workers, sample items were “I feel that my supervisor encourages open communication about safety.” and “I feel comfortable discussing safety issues with my supervisor.” Then, in this study, supplementary items of communication perception (both forward and reverse items) were designed within each subdimension, these descriptions were derived from workers’ viewpoints.

For the exploration of the extent to which the content domain of interest can be reflected by specific set items above, content validity of the initial CWISC scale was evaluated over two rounds. Both item- and scale-level content validity appraisal procedures were executed by a group of six experienced occupational safety experts (Polit and Beck, 2006; Man et al., 2019). The expert panel consisted of one woman and five men, of those, four worked in university, three worked in construction companies. In particular, one had a background in both university and construction company.

#### Analysis

After reviewing the items in first ground, experts were invited to rate each item on its relevance to the affiliated CWISC dimension with four grades (1—not relevant, 2—somewhat relevant, 3—quite relevant, and 4—highly relevant) (Polit and Beck, 2006). Concisely, the score represented its explanatory ability of the default dimension. The item with insufficient agreement on its relevance will be recorded. In second ground, a focus group discussion was implemented to revise items through supplementing, removing, and modifying. These two steps were repeated until all items passed the relevance assessment.

Content validity index (CVI) was the most widely reported approach in scale development studies (Zamanzadeh et al., 2015). According to Polit’s and Beck (2006) recommendation, item-level CVI was calculated as dividing the number of experts giving a rating of either 3 or 4 on one item by the total number of experts, while scale-level CVI was determined by the average of CVI of all items in the scale (Polit and Beck, 2006).

### Results

Based on the literature review and clues from interviews, the layout of total 22 communication scenarios was installed as the initial scale. These items were the description of representative behaviors, they were as general as possible to ensure that workers of various trades were likely to experience them in real-life work environment and they can easily understand the involved situations.

According to the abovementioned rationale and criteria, a content validity assessment was conducted. After eliminating four items that did not pass the assessment process, all 18 retained items had an item-level CVI of 1.0, which led to 1.0 of a scale-level CVI. On these grounds, items contained in the scale had fulfilled the criteria and appeared to be reasonable to measure CWISC. These 18 items displayed in Table 5 were kept for the next stage. Items 1–6 were used to measure CSC, SSC was estimated by items 7–12, and the rest items were prepared for PSC.

**TABLE 5:** Details of 18 items of CWISC.

Construct	Items	Statement	Themes	Source	Scale format
CSC	1	My workmates will accept my advice on safe operation.	N3, N4	Supplementary item from perception	An 5-point phrase completion scale ranging from 1 (indicating “strongly disagree” to 5 (indicating “strongly agree”)
	2	I will tell my workmates how to operate safely when finding him working unsafely.	N3	Meng and Chan, 2020	
	3	I will tell him if I know when my workmates ask me about safety-related issues.	N5	From interview	
	4	My workmates often remind me to pay attention to safety and give me safety information.	N6	Lestari et al., 2020; Deng et al., 2020	
	5	Explaining safety problems to my workmate would delay my work time and schedule.	N3, N4	Supplementary item from perception	
	6	I will remind my workmate to pay attention to safety and give him safety information.	N4	Kincl et al., 2020	
	
SSC	7	When I am not sure about my operation, I will ask my workmates to make sure.	N1	Allison and Kaminsky, 2017	
	8	When I don’t understand the safety signs on site, I will ask my workmates for an explanation.	N2	Kincl et al., 2020	
	9	In order to ensure my personal safety, I will confirm whether my behavior is safe with my workmates during the construction operation.	N2	From interview	
	10	My workmates is willing to answer my safety questions.	N2	Supplementary item from perception	
	11	I can freely ask questions to my workmates if I have safety problems.	N1	From interview	
	12	I find it troublesome to ask workmates for safety help and explanations.	N2	Supplementary item from perception	
	
PSC	13	I feel comfortable discussing safety issues with my workmates.	N8	Supplementary item from perception (Zhang et al., 2020)	
	14	I would like to share my safety operation experience with my workmates.	N9	Li et al., 2020	
	15	I often discuss safety issues with my workmates when taking a break on site.	N8	Deng et al., 2020	
	16	When my workmates discussing construction safety issues, I will actively participate.	N7	From interview	
	17	I am free to speak up when discussing safety issues with my workmates.	N9	Chen, 2017	
	18	Getting involved in safety discussions is a waste of my time.	N8	Supplementary item from perception	

## Study 3

### Methods

#### Participants

The data used in EFA were collected from questionnaire surveys. In the formal distribution from May 2021 to July 2021, questionnaires were dispensed to a total of 300 construction workers from high-rise residential building projects, several cities in northern (Harbin and Shenyang), central (Jinan and Handan), and southern (Guiyang and Guangzhou) China were selected for investigation. Participations were voluntary and not required to provide any personal or identifiable information in the questionnaire.

#### Procedure

Before the formal investigation, a pilot test was performed for several selected workers. Questions were further simplified and rephrased to be much clear for workers with limited education. The final questionnaire consisted of two parts: measurement items and general demographic questions, including gender, age, years of work experience, education level, and trade types. Senior managers from the project emphasized the academic purpose of research to participants, and this support facilitated the development of investigation. Meanwhile, to ensure the quality of reply, workers who could not fully understand the statements completed it with the help of researchers. Finally, after removing incomplete replies as well as answers with high repetition, a total of 219 valid responses were used in the data analysis process (a response rate of 73%).

#### Data Analysis

Using Statistical Package for the Social Science (SPSS) 25, EFA was performed to render the number of latent variables based on commonalities and further inspect factor loadings of items. EFA is a statistical technique extensively applied in scale development to explore underlying dimensions of measurement items. Considering that there were a few references for multidimensional constructs of CWISC at the present stage, this study expects to maximize the use of survey data to explore objective laws. Principal component analysis (PCA), an adaptive exploratory approach for data processing, compression, and extraction, has been widely used in scale development studies. Through PCA, the cumulative explanation of extracted principal component (factor) to total variance can be maximized (Jolliffe and Cadima, 2016). This principle of factor extraction is consistent with the expectation of minimizing data losses in this study. Simultaneously, PCA is highly inclusive for data types, there is no need to make any assumptions about the prior distribution of evaluation data, e.g., normal distribution (Walker, 2010). Accordingly, the PCA method with an oblique rotation was preferred. Instead of commonly used orthogonal rotation, an oblique rotation is theoretically more accurate in the context of social science research in which correlations among factors are generally expected (Jian et al., 2014). On account of this, it is unreasonable to assume items to be completely uncorrelated to each other.

Sampling adequacy for EFA was assessed by the Kaiser–Meyer–Olkin (KMO) test and the value of *p*, with the criteria to be >0.50 and <0.01, respectively (Zhou et al., 2021). Equally, items with factor loading < 0.5 and loaded on the factors other than its design will be dropped out of scale. The accumulated variance explanation by extracted factors was expected to exceed 60% while Cronbach’s alpha should be more than 0.70.

### Results

#### Descriptive Analyses

**FIGURE 3:**
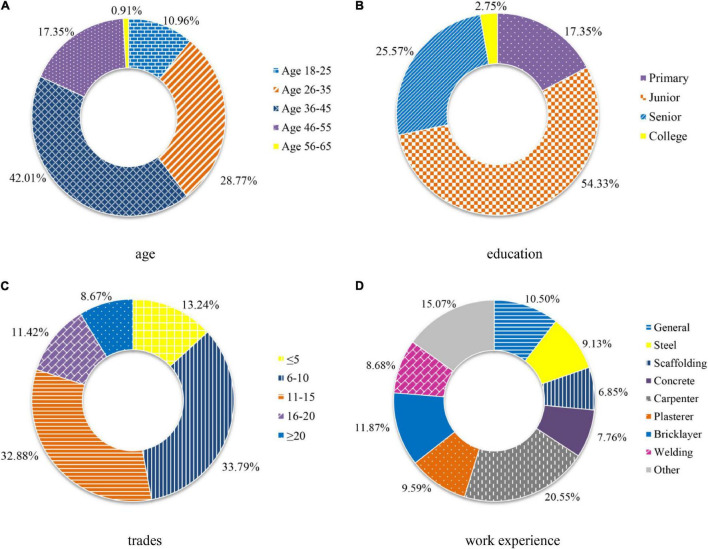
Demographic profiles of workers participated in the exploratory factor analysis (EFA) in terms of **(A)** age, **(B)** education, **(C)** trades, and **(D)** work experience.

According to the statistical analysis on valid respondents, respondents were mostly men (97.26%) due to the men-dominant workforce in the Chinese construction industry. Other demographic information was shown in Figure 3. The majority of respondents received at least junior school education (82.65%), and they had more than 5 years of work experience in the construction industry (86.76%).

#### Exploratory Factor Analysis

Following steps were performed firstly before the factor extraction process, including numerical conversion of reverse items, recording and scoring reverse items, and assigning new values to old ones. Then, the KMO and Bartlett test were examined to analyze a correlation between original variables, namely whether it was suitable for the factor analysis (Clark and Watson, 1995). The results showed that the KMO value was 0.902 and the significant level of Bartlett test was 0, it indicated an appropriateness of data for the factor analysis.

**FIGURE 4:**
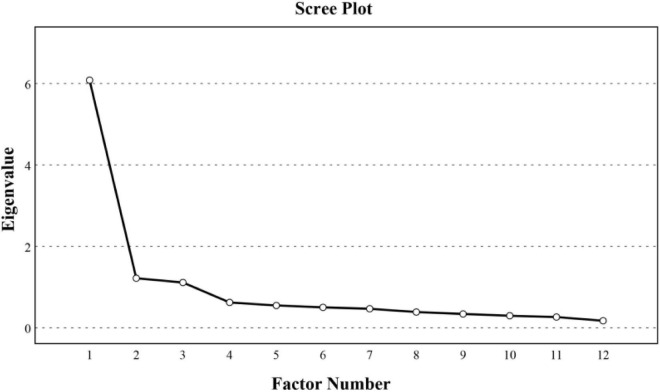
A scree plot for the number of factors to be retained.

The scree test and eigenvalue (>1) recommended by Costello and Osborne (2005) were used in combination to decide the number of factors to be extracted (Costello and Osborne, 2005). The graph of eigenvalues in the scree test (Figure 4) was studied, and the break point of data where the curve flattened out was identified. Finally, three factors were clearly identified with the consideration of the criteria mentioned; the result was in accordance with the conceptual framework proposed in stage 1. Accordingly, a total of 12 items of the CWISC scale were retained for further analysis after removing six unbefitting items. For the clarity of presentation, factor loadings below 0.3 were not shown in Table 6 as done in the study of Man et al. (2019). The factor loadings of the 12 items belonging to three dimensions varied from 0.519 to 0.920, and each item had a unique contribution to one of these three factors. The eigenvalues of CSC, SSC, and PSC were 6.081, 1.216, and 1.111, respectively, these dimensions accounted for 70.075% of the total variance.

**TABLE 6:** The results of the exploratory factor analysis (EFA).

Item	content	Factor loading			Communality
		SSC	OCSC	PSC	
1	SSC1	0.920			0.787
2	SSC2	0.854			0.760
3	SSC4	0.823			0.711
4	SSC5	0.772			0.753
5	SSC6	0.790			0.646
6	CSC2		0.787		0.649
7	CSC3		0.854		0.648
8	CSC4		0.818		0.669
9	CSC6		0.701		0.573
10	PSC1			0.519	0.724
11	PSC2			0.610	0.717
12	PSC4			0.919	0.772
Eigenvalue cumulative %		6.081	1.216	1.111	
of explanatory variance		50.679	60.815	70.075	

The reliability and validity of the scale were tested to demonstrate their effectiveness. Cronbach coefficient was used to measure the reliability of the questionnaire. In this stage, the overall Cronbach’s α of the scale with 12 items was 0.910, the three subscales also obtained excellent internal consistency following that a Cronbach’s alpha value above 0.70 was recommended to ensure data reliability (Kim et al., 2021). Cronbach’s α of CSC, SSC, and PSC reached 0.810, 0.902, and 0.770. As consequence, all 12 items derived from EFA were worth retaining to the next stage of scale validation.

## Study 4

### Methods

#### Participants and Procedure

**FIGURE 5:**
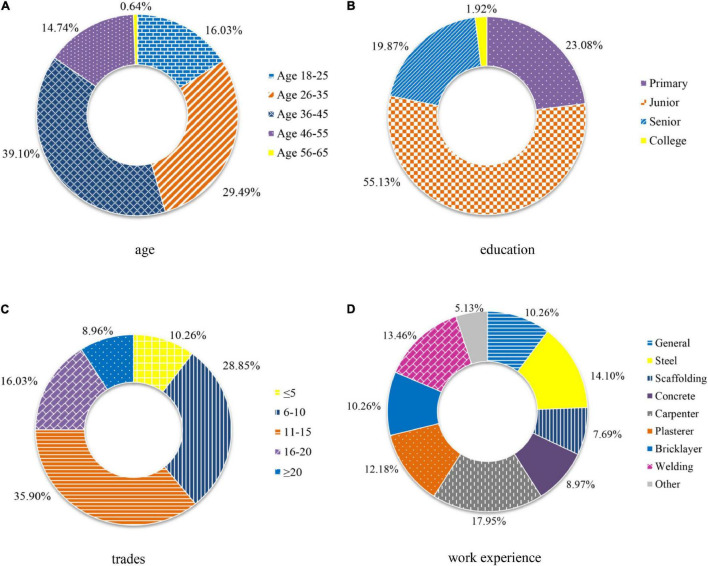
Demographic profiles of workers participated in the confirmatory factor analysis (CFA) in terms of **(A)** age, **(B)** education, **(C)** trades, and **(D)** work experience.

For the verification of item-factor relationships (factor loadings) and the underlying dimensions of the instrument, CFA, one type of structural equation modeling (SEM) (Ullma, 2006), was conducted with a maximum likelihood method. Based on another sample of 156 construction workers, the factor structure derived from stage 3 was incorporated as a measurement model in CFA. This process played an important role in validating the hypothesized model and inspecting the reliability of the measurement. The location of the investigated cities was the same as that in stage 2, and identical distribution and scoring criteria of the questionnaire were employed in this stage. The statistical analysis of workers was depicted in Figure 5 with the same color and fill formats as Figure 3. There was no significant difference between the samples used in the two stages, since fluctuations for the proportion of each part were within 6% (Xie et al., 2021).

#### Data Analysis

Followed Crawford’s and Kelder’s suggestions of model fit indices, we reported the Chi-square value (χ^2^), root mean square error of approximation (RMSEA), comparative fit index (CFI), Tucker–Lewis’s goodness-of-fit index (TLI), and standardized root mean square residual (SRMR) to indicate the model-data fit (Hooper et al., 2008; Kline, 2011). Cronbach’s alpha for internal consistency reliability, composite reliability (C.R.), convergent validity, and discriminant validity were also assessed.

### Results

#### Confirmatory Factor Analysis

**FIGURE 6:**
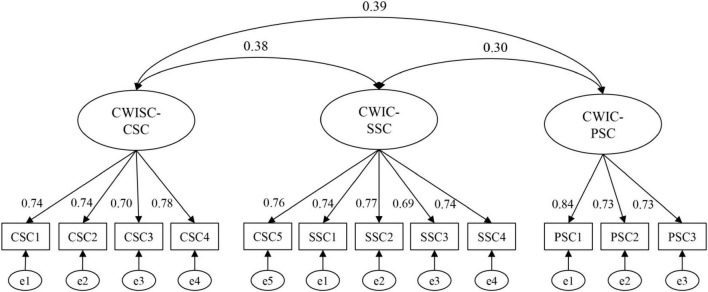
Three-factor measurement model of CWISC with standardized estimates on arrows.

The three-factor measurement model was tested as illustrated in Figure 6. The results revealed a good fit to data. The goodness-of-fit indices were adequate with χ^2^ = 68.738, χ^2^/*df* = 1.348, *p* = 0.049, CFI = 0.976, TLI = 0.968, RMSEA = 0.047, standardized RMR = 0.050, GFI = 0.930, and IFI = 0.976. The values of these goodness-of-fit statistics demonstrated an acceptable model fit (McDonald and Ho, 2002; Kline, 2011).

#### Reliability Assessment

Given that sufficient evidence for the integrity of the factor structure has emerged, its reliability was then assessed. Internal consistency reliability was used to describe the extent to which all items in the scale measured the same concept or construct (Tavakol and Dennick, 2011). With a criterion level of 0.7, Cronbach’s alpha was employed to measure internal consistency reliability of the scale (Man et al., 2019). The overall internal reliability of CWISC scale was 0.837, Cronbach’s α of subscales and C.R. were shown in Table 7. The results showed a range from 0.807 to 0.856 for Cronbach’s α of subscales, and a range from 0.810 to 0.858 of C.R., these indicated an acceptable consistency reliability as well as CR.

**TABLE 7:** Reliability and descriptive statistics of the CWISC scale.

Factor	Item	Factor loading	Cronbach’s α	Composite reliability (C.R.)	AVE	Square roots of AVE	*M*	*SD*
SSC			0.856	0.858	0.547	0.740		
	SSC1	0.762					3.62	0.854
	SSC2	0.740					3.69	0.948
	SSC3	0.767					3.46	0.973
	SSC4	0.687					3.43	0.978
	SSC5	0.739					3.48	0.898
CSC			0.829	0.830	0.549	0.741		
	OCSC1	0.736					3.31	0.989
	OCSC2	0.744					3.29	0.979
	OCSC3	0.700					3.22	0.920
	OCSC4	0.782					3.29	0.937
PSC			0.807	0.810	0.589	0.767		
	PSC1	0.836					3.44	1.036
	PSC2	0.732					3.52	0.974
	PSC3	0.729					3.50	0.933

#### Validity Assessment

The final stage involved testing for convergent and discriminant validity of the final version of the CWISC scale. Convergent validity, which refers to a correlation between two or more scores on the scale, is designed to assess similar constructs (Chen and Tung, 2014). It can be determined by factor loadings and the average variance extracted (AVE) value for every construct (Fornell and Larcker, 1981). As shown in Table 7 and Figure 6, factor loadings of observed variables were significant between 0.69 and 0.84, the values of AVE were all greater than 0.5. Those statistics illustrated that the convergent validity of the CWISC scale was acceptable (Fornell and Larcker, 1981).

Discriminant validity refers to the extent to which the measure is indeed novel and not simply a reflection of few other variables (Churchill, 1979). The square root of the AVE value was adopted to assess the discriminant validity of the CWISC scale. As shown in Tables 7, 8, all square roots of AVE were greater than construct correlations, which implied discriminant validity to be acceptable (Fornell and Larcker, 1981). Ultimately, based on the processes presented earlier, the final CWISC scale with 12 items was generated.

**TABLE 8:** Correlations among constructs.

Constructs	SSC	CSC	PSC
SSC	1.000		
CSC	0.376***	1.000	
PSC	0.299**	0.388***	1.000

**p < 0.01; ***p < 0.001.

## Discussion

The prevalence of CWISC on construction sites and its limited understanding called for the conceptualization and quantification of a construct. Thus, this study presented the development and validation process of the CWISC scale in terms of its ability to measure the communicational characteristics, which were not easy to be detected and rewarded on construction sites. The final scale contained 12 items and three dimensions: CSC, SSC, and PSC. The obtained results indicated that the CWISC had a clear factor structure and adequate metric properties with good validity and reliability, the finding prepared an instrument to aid in understanding and estimating informal safety communication of workers on construction sites.

Construction workers’ informal safety communication demonstrated workers’ different types of informal safety communication under multiple motivations. As the first dimension, the measurement items of CSC reflected altruistic safety communication in both proactive and passive situations. For instance, proactive CSC was described by workers as “I will tell my workmates how to operate safely when finding him working unsafely,” while passive CSC was embodied as “I will tell him if I know when my workmates asking me about safety-related issues.” The proposal of proactive CSC was essentially in accordance with the measurement items involved in safety climate (Gao et al., 2017; Zhang et al., 2020) and SCB (Meng and Chan, 2020). As described about “interpersonal helping” in Table 2, those descriptions were all actions of initiating CSC. Moreover, the study further reported the passive CSC, which was proved to be another form of participation in altruism-actuated safety communication. This implied that when a worker was asked by coworkers for safety information, the altruistic motivation would facilitate him to respond, it contributed to create a virtuous cycle of SSC–CSC. Conversely, inability to obtain the required safety information will impair the willingness of workers to ask a safety-related question. Considering the discretion of CSC, the results also led to the need to explore incentives, even beyond the formal reward system, to increase workers’ positivity to join CSC.

The second dimension, SSC, was closely linked to workers’ own safety needs, which can be well explained by Maslow’s hierarchy of needs. The theory classified human needs into five grades from low to high, those were physiological needs, safety needs, belonging, esteem, and self-actualization (Maslow, 1943). It described the process of individual’s demand chasing, in which the realization of lower-level needs was a prerequisite for individuals to pursue needs at a higher level (Wahba and Bridwell, 1976; Hale et al., 2019). As identified in the second level, safety needs of construction workers was deemed to be primary, only when a worker fulfilled the expected safety requirements for personal and workplace safety, can other subsequent needs be pursued. Despite the fundamental safety needs have pushed SSC to the forefront, it was only mentioned in limited studies as the annotation in Table 2 “Calling friends on how to handle a work problem” (Allison and Kaminsky, 2017; Chen et al., 2021). More attention needs to be paid considering its priority among three subdimensions of CWISC.

Apparently, in view of the highest mean in three subdimensions and the support from the fundamental safety needs, SSC was evidenced to be the most basic form of safety communication. Moreover, SSC accounted for 50.68% of the total variance, which also indicated that it was a dominant factor. The opinion that workers were considered to be responsible for their own safety was in line with Didla’s exposition. In the course of his interviews, workers claimed that it was their duty to keep their own safety, they did not want to be hurt (Didla et al., 2009). However, nearly half of the workers interviewed in this study admitted that they hardly ever asked coworkers for safety information. When ambiguous safety information emerged at work, they felt feasible to follow their own work experiences, this tendency may put themselves into a dangerous situation. Being the least mentioned event during interviews (15), as well as receiving an unexpected low mean score (below 0.4) in the investigation, an urgent need to give particular concerns on exploring the underlying reasons behind workers’ experience-preferred operation is also stressed. A cognitive failure of a worker, which is caused by ambiguity or lacking of indispensable safety information, will ultimately lead to the occurrence of unsafe behaviors (Fang et al., 2016).

For the third dimension, PSC and the covered items were defined to evaluate workers’ representation on safety information sharing. The measurement items revolving around safety discussion were designed based on existing research, as “I feel comfortable discussing safety issues with my workmates” and “I would like to share my safety operation experience with my workmates.” Meanwhile, the result provided a well explanation for the dynamic discussion process under Cognitive Surplus and Herd Mentality. In the state of Cognitive Surplus, experienced workers are willing to share their safety-related knowledge in their rest time, this action was considered to be an important channel for the transmission of tacit knowledge within the crew (Matei, 2012). Additionally, unlike workers who initially engaged in safety discussion with the mindset of sharing information, the participation of some workers was detected to be an efficacy of Herd Mentality. Some workers declared that they joined in the discussion initially just because familiar workers were involved. With the enthusiasm and openness of the discussion gradually increased, they became willing to share their opinions. This phenomenon of conformity was in line with the inspiration from Liang’s research, in which workers’ safety violations were shown to be contagious within the crew (Liang et al., 2018). This led to the consideration on how to promote the positive effect of Herd Mentality through management interventions. In view of that, open safety discussion provides a shared understanding of expected behaviors and how procedures should be translated into work practices (Michael et al., 2006), efforts should be made to facilitate workers’ willing to participate in safety discussion as well as their positive attitude toward PSC.

All three types of CWISC can promote transferring and sharing of safety-related information within crews, they also contribute to the cultivation of risk management capability together with safety awareness of workers. The dynamic process of safety communication produces an important access to achieve self-safety management within the workers group, it further elicits the formation of a worker-centered adaptive system, by which the proactive safety management was expected to achieve. The development and cultivation of proactive self-management should be emphasized and applied in combination with passive management methods, such as the implementation of management interventions. The study is expected to draw attention to the importance of CWISC in follow-up studies.

## Theoretical Implications

The CWISC framework and the corresponding scale developed in this article are expected to generate the following important theoretical implications. First, the findings of this study reflected the applicability of relevant theories in construction safety research, these provided new explanations for intra-team safety communication. Then, this study was an extension of previous research investigating the communication network in a safety context to increase our understanding on its composition. Following the basic attributes of organizational communication, such as the direction of information transmission and communication channels, the findings provided supportive evidence on the three types of CWISC from the perspective of individual motivation. In particular, a valuable source of information formed by the study provided richer insights into CWISC, these findings had a potential to highlight a disparity within these dimensions. Finally, an in-depth analysis of the construct was helpful to improve the theories of safety communication under the construction background. Theoretically, the CWISC scale, as a multi-tested measurement tool, was a necessary basis for derivative research. It is expected to contribute to the completion of more research focusing on safety communication among workers.

## Practical Implications

Apart from theoretical implications, this research also generated the following practical implications. Initially, through the adoption of the proposed CWISC scale, managerial benefits may be obtained in developing and implementing effective safety management strategies. The scale can serve as an instrument to assess the performances and tendency of workers’ safety communication, the results may help in identifying weaknesses in safety communication. Consequently, wasted management costs could be avoided by selecting and implementing the targeted incentive programs. Further, the practice issue of poor performances within workers’ responsibilities was identified and highlighted. The authors believe that there are a mixed set of reasons for the poor SSC on construction sites, such information will hopefully increase the attention of managers and researchers to the problem. Finally, the management intervention of setting up model workers to inspire coworkers’ passions for safe communication is recommended to be carried out. It is expected that the CWISC scale could lead to a diverse approach in practice to authenticate and augment workers’ safety communication performances.

## Limitations and Future Research

Despite contributions of the proposed CWISC framework and its corresponding scale, its limitations must be recognized and future research should be conducted. Although the CWISC scale is expected to have applicability across different countries, the findings of this study were limited to the construction industry in China, it is necessary to further verify its validity in other countries. Then, given the difficulty and limitation of data acquisition, 219 samples and 156 samples were used in EFA and CFA, respectively; a larger sample size should be employed in follow-up studies to validate the developed scale. In addition, there are many sociodemographic factors (e.g., education, work experience, and trade) that could affect safety communication behaviors. It is worthwhile to expand research in this area to obtain a full picture of the CWISC phenomenon. Finally, the study gave a particular focus on CWISC itself to dissect its classification and relative characteristics, efforts should be made in future studies to explore its facilitators and inhibitors. The predictability of CWISC on safety culture or other indicators of safety performance is also suggested for examination. Considering the important role of CWISC on safety performance in the context of construction, abundant research will provide valuable references for the development of management measures.

## Conclusion

Construction workers’ informal safety communication has always been unavoidable on construction sites. By conducting a four-phase deductive–inductive study with a qualitative and quantitative analysis, a comprehensive measurement instrument was developed and validated, the CWISC scale with 12 items was designed to assess workers’ communication performances on construction sites. In addition, three dimensions of CWISC, CSC, SSC, and PSC, were identified. Given the lack of concern on exploring the framework and measurement scale of CWISC, it may provide a distinct contribution to theory building and assessment practice on intra-crew safety communication. Moreover, the theoretical and practical implications will draw the attention of managers and researchers to consider the management issues related to it. Despite the useful results of this study, additional works should be carried out to further validate the scale under different backgrounds; its limitations should also be addressed in the future. We expect that the study can lead to diverse research in which the three-dimensional construct of CWISC will be taken into account.

## Data Availability Statement

The raw data supporting the conclusions of this article will be made available by the authors, without undue reservation.

## Ethics Statement

Ethical review and approval was not required for the study on human participants in accordance with the local legislation and institutional requirements. Written informed consent for participation was not required for this study in accordance with the national legislation and the institutional requirements.

## Author Contributions

WC and YS put forward the research idea and promoted the implementation and completion of this research. SZ, HL, and HX supported the data collection. WC involved in statistical analysis, the interpretation of the results, and composing the first draft. HL and HX gave help to further optimize and complete this manuscript. All authors contributed to this article and approved the submitted version.

## Conflict of Interest

The authors declare that the research was conducted in the absence of any commercial or financial relationships that could be construed as a potential conflict of interest.

## Publisher’s Note

All claims expressed in this article are solely those of the authors and do not necessarily represent those of their affiliated organizations, or those of the publisher, the editors and the reviewers. Any product that may be evaluated in this article, or claim that may be made by its manufacturer, is not guaranteed or endorsed by the publisher.
